# Tweaking Progranulin Expression: Therapeutic Avenues and Opportunities

**DOI:** 10.3389/fnmol.2021.713031

**Published:** 2021-07-23

**Authors:** Joke Terryn, Catherine M. Verfaillie, Philip Van Damme

**Affiliations:** ^1^Department of Neurosciences, Experimental Neurology and Leuven Brain Institute (LBI), KU Leuven-University of Leuven, Leuven, Belgium; ^2^Laboratory of Neurobiology, Center for Brain and Disease Research, VIB, Leuven, Belgium; ^3^Department of Development and Regeneration, Interdepartmental Stem Cell Institute, KU Leuven-University of Leuven, Leuven, Belgium; ^4^Department of Neurology, University Hospitals Leuven, Leuven, Belgium

**Keywords:** progranulin (GRN), frontotemporal dementia (FTD), frontotemporal lobar degeneration (FTLD), amyotrophic lateral sclerosis (ALS), neurodegeneration, gene therapy, therapeutic target

## Abstract

Frontotemporal dementia (FTD) is a neurodegenerative disease, leading to behavioral changes and language difficulties. Heterozygous loss-of-function mutations in *progranulin* (*GRN*) induce haploinsufficiency of the protein and are associated with up to one-third of all genetic FTD cases worldwide. While the loss of GRN is primarily associated with neurodegeneration, the biological functions of the secreted growth factor-like protein are more diverse, ranging from wound healing, inflammation, vasculogenesis, and metabolic regulation to tumor cell growth and metastasis. To date, no disease-modifying treatments exist for FTD, but different therapeutic approaches to boost GRN levels in the central nervous system are currently being developed (including AAV-mediated* GRN* gene delivery as well as anti-SORT1 antibody therapy). In this review, we provide an overview of the multifaceted regulation of GRN levels and the corresponding therapeutic avenues. We discuss the opportunities, advantages, and potential drawbacks of the diverse approaches. Additionally, we highlight the therapeutic potential of elevating GRN levels beyond patients with loss-of-function mutations in *GRN*.

## Introduction

### FTD-ALS Spectrum Disorders

Frontotemporal dementia (FTD) is a neurodegenerative disease, leading to behavioral changes and language difficulties. Previously considered a rare disease entity, FTD is in fact the third leading cause of all dementia cases (Rabinovici and Miller, 2010). FTD typically presents before the age of 65 and is therefore considered a presenile dementia. In this age group (<65), FTD is as common as Alzheimer’s dementia (Rabinovici and Miller, 2010; Ferrari et al., 2014). The prevalence of FTD varies regionally (ranging from 3 to 15 per 100,000 in the 45- to 64-year-old population; Rabinovici and Miller, 2010). In contrast to other types of dementia, FTD patients have a positive family history in 30–40 percent of all cases, with mutations in three genes (*GRN*, *MAPT*, and *C9orf72*) explaining the majority of the genetic cases (Moore et al., 2020).

The term FTD is used to bundle the spectrum of clinical manifestations, while frontotemporal lobar degeneration (FTLD) refers to the underlying neuropathology. FTLD neuropathological subtypes were established based on the proteins found in the neuropathological inclusions. The two main subtypes are FTLD-tau for microtubule-associated protein tau (*MAPT*) positive inclusions, and FTLD-TDP/FTD-U for ubiquitin and transactive response DNA-binding protein 43 (TDP-43) positive inclusions (Mackenzie et al., 2006).

The first FTD gene was discovered in 1998, when mutations in *MAPT* were identified in some families with FTD and Parkinsonism. Surprisingly, some of the cases linked to the same chromosomal locus (17q21) were tau-negative. In 2006, mutations in the neighboring *GRN* gene were discovered, explaining the genetic linkage to the same chromosomal locus (Baker et al., 2006; Cruts et al., 2006). The neuronal inclusions found in these patients were not GRN immunoreactive and *GRN* mutations were shown to lead to reduced *GRN* expression (Baker et al., 2006; Cruts et al., 2006). Shortly after the discovery of the *GRN* gene, TDP-43 was identified as the key constituent of the tau-negative neuronal inclusions (Neumann et al., 2006). As FTLD-TDP pathology is responsible for 45% of FTD cases and the majority of amyotrophic lateral sclerosis (ALS) cases, the finding consolidated the link between FTD and ALS (Ling et al., 2013; Tziortzouda et al., 2021). In 2011, the discovery of a hexanucleotide (GGGGCC) repeat expansion mutation in *C9orf72* finally explained the genetic link to the ninth chromosome in cases with ALS and FTD (DeJesus-Hernandez et al., 2011; Renton et al., 2011). Repeat expansions in *C9orf72* are the most common genetic cause of FTD and ALS. Other, more rare mutations, such as mutations in charged multivesicular body protein 2B (*CHMP2B*), Valosin-Containing Protein (*VCP*), ubiquilin 2 (*UBQLN2*), TANK-Binding Kinase 1 (*TBK1*), sequestosome 1 (*SQSTM1*), and T cell–restricted intracellular antigen 1 (*TIA1*) explain residual cases of genetic FTD (Pottier et al., 2016).

Behavioral variant frontotemporal dementia (bvFTD) is the most common clinical manifestation of frontotemporal lobar degeneration, occurring in 60 percent of cases. When language is primarily impaired the term primary progressive aphasia is used (PPA) (Rascovsky and Grossman, 2013). Generally, three variants of PPA are described—the semantic, logopenic and nonfluent/agrammatic variant—but mixed variants exist (Gorno-Tempini et al., 2011; Vandenberghe, 2016). In addition to the typical manifestations (bvFTD and PPA), other disorders have been linked to the FTD spectrum: Frontotemporal Dementia with Motor Neuron Disease (FTD-MND), corticobasal degeneration, and progressive supranuclear palsy (Olney et al., 2017).

### The *GRN* Gene

In humans, the *progranulin (GRN)* gene is located on chromosome 17q21, 1.7 Mb centromeric of *MAPT*, and consists of 12 protein-coding exons and a 5′ non-coding exon. The mammalian *GRN* gene codes for a repetition of seven and a half granulin domains. Each granulin domain is encoded by two neighboring exons, contributing either the N- or C-terminal half of the domain. The unique structure of the evolutionary conserved granulin motif is reviewed in Palfree et al. (2015).

Since the discovery in 2006 of *GRN* mutations linked to FTD (Baker et al., 2006; Cruts et al., 2006), over 130 pathological mutations in the *GRN* gene have been described (Moore et al., 2020). The majority of *GRN* mutations are nonsense and frameshift mutations, that introduce premature stop codons and result in nuclear degradation of the mutant mRNA, suggesting GRN haploinsufficiency underlying *GRN*-linked neurodegeneration (Yu et al., 2010; Kleinberger et al., 2013). Other mutations in the growing list of *GRN* mutations, result in deletion of the gene (Gijselinck et al., 2008), affect the initiation of translation (Le Ber et al., 2008), processing (Shankaran et al., 2008), and secretion (Mukherjee et al., 2006). Several studies showed that patients with a *GRN* mutation have reduced GRN protein levels in the cerebrospinal fluid (Ghidoni et al., 2008; Van Damme et al., 2008). Similarly, a reduction of blood GRN levels can be seen, which can be used to predict the presence of a pathogenic *GRN* mutation (Finch et al., 2009).

The phenotypic variability seen in patients with *GRN* mutation is astonishing (Moore et al., 2020). Behavioral variant frontotemporal dementia and nonfluent/agrammatic variant PPA are the most common diagnoses in this genetic group (Kim et al., 2013). Mild Parkinsonism is a common clinical finding in *GRN* mutation carriers (Le Ber et al., 2008). Clinical presentations indistinguishable from typical Alzheimer’s or Parkinson’s disease are occasionally seen (Brouwers et al., 2007). Rarely, GRN mutations present as motor neuron disease (Benussi et al., 2009), corticobasal syndrome or progressive supranuclear palsy (Baizabal-Carvallo and Jankovic, 2016).

### The Pleiotropic GRN Protein

When GRN entered the neurodegenerative scene, the protein was already known as a widely expressed growth factor. The GRN protein had been identified by a number of independent research groups in different biological contexts and is therefore known by many names. Sequencing revealed the shared genetic origin of proepithelin (Plowman et al., 1992), granulin-epithelin precursor (GEP) (Zanocco-Marani et al., 1999), PC cell derived growth factor (PCCDGF) (Zhou et al., 1993), and acrogranin (Baba et al., 1993). A multitude of names underscore the pleiotrophy of the GRN protein.

Initial work was focused on the mitogenic and tumor-promoting effects of GRN. High GRN expression has been detected in many types of cancer and elevated serum GRN levels could be used as a potential prognostic biomarker in for example breast cancer, chronic lymphocytic leukemia, and non-small-cell lung carcinoma (Arechavaleta-Velasco et al., 2017). GRN seems to stimulate tumor growth and promotes tumor cell migration, invasiveness, anchorage independence, and chemo-resistance (He and Bateman, 1999; Bandey et al., 2015; Tanimoto et al., 2016). Important to note, however, is that while GRN stimulates tumor progression, it does not induce malignant transformation on its own. GRN however, can influence the transition from a precancerous state to a highly tumorigenic state (Matsumura et al., 2006). As GRN was shown to be an important therapeutic and diagnostic target in breast cancer, GRN diagnostic kits and neutralizing antibodies are being developed (Guha et al., 2021).

**Figure 1:**
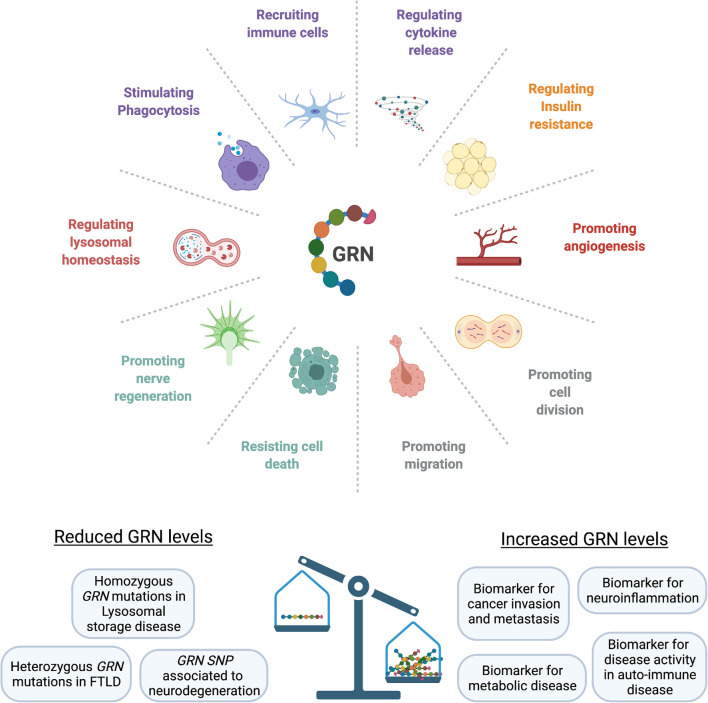
GRN affects multiple biological functions. At physiological levels, GRN is involved in development, fine-tuning the immune response, orchestrating tissue repair, and providing neurotrophic support. Loss of progranulin can lead to an uncontrolled inflammatory response, loss of lysosomal homeostasis, and neuronal death. GRN levels are elevated in cancer, metabolic diseases, and chronic inflammatory states (SNP, single-nucleotide polymorphism; FTLD, Frontotemporal lobar degeneration).

Endogenous GRN expression is found throughout the body, with high expression levels in neural tissue, immune cells and epithelia lining the reproductive organs, the gastrointestinal tract, and the skin (Daniel et al., 2000). GRN is closely involved in embryogenesis, but its expression becomes more restricted later in development (Bateman and Bennett, 2009). At physiological levels, GRN is typically expressed under conditions of tissue remodeling and repair (Bateman and Bennett, 2009), where it exerts multifaceted roles. For example in wound healing, GRN stimulates migration and mitosis of fibroblast and endothelial cells, promotes blood vessel formation, and controls the recruitment and activity of leukocytes (He et al., 2003). In adipose tissue, GRN secretion correlates with macrophage infiltration (Youn et al., 2009) and GRN was found to be a key regulator of insulin sensitivity (Matsubara et al., 2012; Zhou B. et al., 2015). Because GRN expression is increased in acute and chronic inflammatory states, GRN has been proposed as a biomarker for diseases with a strong inflammatory component, including metabolic, cardiovascular, and auto-immune diseases (Abella et al., 2017). Spontaneously occurring neutralizing GRN antibodies have been detected in patient’s sera in association with multiple auto-immune diseases (Thurner et al., 2013), suggesting an important function of GRN as a modulator of the immune response. Figure 1 summarizes the diverse biological roles of GRN.

#### Role of GRN in the Central Nervous System (CNS)

Because *G*RN had been known as a widely expressed growth factor with important roles in development, inflammation, and tumorigenesis (He and Bateman, 2003), the discovery of GRN mutations in an adult onset neurodegenerative disease was surprising. GRN haploinsufficiency in the brain, as seen in FTD cases with *GRN* mutations, leads to chronic degenerative changes of neuronal loss, gliosis, and microglial activation (Mackenzie et al., 2006). Because GRN expression is restricted to neurons and microglia in the central nervous system (CNS) (Daniel et al., 2000; Petkau et al., 2010), both celltypes have been postulated as drivers of neuropathology in *GRN-*FTLD cases.

Proof for the direct neurotrophic effect of GRN was provided when extracellular administration of GRN was shown to stimulate neurite outgrowth and survival in cultured primary cortical and motor neurons (Van Damme et al., 2008; Gao et al., 2010). Likewise, neurons derived from *Grn* null mice were more susceptible to toxic insults (Kleinberger et al., 2010) and showed reduced neurite outgrowth and branching (Gass et al., 2012; Beel et al., 2017). While studies in multiple model systems have supported a direct role for GRN in neuronal development and survival (Kleinberger et al., 2013), the mechanisms by which GRN functions and how the shortage of GRN causes TDP-43 pathology and neurodegeneration, is only partially understood.

In addition to the growth factor properties, the immune modulatory role of GRN has been explored in the CNS as well. A prominent feature of *Grn* null mice is the consistent age-dependent microgliosis and astrogliosis (Ahmed et al., 2010; Yin et al., 2010). Mouse *Grn*-deficient microglia were shown to express higher levels of proinflammatory cytokines and contributed to neuronal death (Martens et al., 2012). Enhanced phagocytosis in response to the loss of GRN has been demonstrated in multiple animal models. Nematodes lacking *pgrn-1* appeared normal, but exhibit fewer apoptotic cell corpses during development, due to rapid clearance of apoptotic cells (Kao et al., 2011). An increased appetite for inhibitory synapses was demonstrated in *Grn* null microglia and was shown to be associated with compulsive grooming behavior (Lui et al., 2016). These findings could indicate that GRN normally suppresses microglial activity and that excessive clearance upon loss of GRN could prevent normal neuronal recovery.

While many studies point to microglia as drivers of pathology, a number of conditional knockout studies nuance this hypothesis. Selective depletion of either neuronal or microglial *Grn* was shown to produce different behavioral deficits. While depletion of neuronal *Grn* provoked social dominance defects (Arrant et al., 2017, 2019), depletion of microglial *Grn* triggered compulsive behaviors (e.g., excessive grooming) (Krabbe et al., 2017; Arrant et al., 2019). Interestingly, neither the combined nor isolated depletion of neuronal or microglial *Grn* was sufficient to produce gliosis and lipofuscinosis in mice (Petkau et al., 2017; Arrant et al., 2019). Seemingly, low residual doses of *Grn* are sufficient to prevent gliosis and lipofuscinosis. This is in analogy with the lack of obvious neuropathological changes in heterozygous *Grn* mutant mice (Ahmed et al., 2010). In addition to the selective depletion studies focusing on the CNS, a study examined the relative contribution of microglial and neuronal *Grn* on the recovery from facial nerve injury (Beel et al., 2017). The study showed that neuronal *Grn* and not microglial *Grn* is essential for nerve regeneration.

Given that different cell types provoke different behavioral deficits (Krabbe et al., 2017; Arrant et al., 2019) and different mechanisms seem to be at play in neurons and microglia (Lui et al., 2016; Beel et al., 2017; Krabbe et al., 2017), combined targeting of both neurons and microglia might be needed to tackle different aspects of *GRN*-linked neurodegeneration.

#### Role of GRN in Lysosomal Storage Disease

A very curious case report in 2012 redirected the GRN field towards the lysome (Smith et al., 2012). Exome sequencing of two Italian siblings with neuronal ceroid lipofuscinosis (NCL) revealed that homozygous loss of *GRN* caused the lysosomal storage disease (Smith et al., 2012). The clinical picture of the siblings resembled in no apparent way a more severe manifestation of FTD. The sisters had developed generalized epilepsy in their early twenties, combined with retinal dystrophy, cerebellar ataxia, and cognitive deterioration. Brain MRI showed focal atrophy of the cerebellum, not of the frontal and temporal lobes. Skin biopsies were taken as the clinical picture of progressive dementia, visual loss, epilepsy, and motor deterioration was suggestive for neuronal ceroid lipofuscinosis (NCL). Electron microscopic examination confirmed the presence of typical fingerprint profiles in endothelium and secretory cells, diagnostic of NCL. Consequently, the *GRN* gene joined the list of NCL genes as CLN11 (Smith et al., 2012). The neuronal ceroid lipofucinoses are, as a group, defined by selective neurodegeneration and the deposition of autofluorescent storage material (either subunit c of mitochondrial ATP synthase or saposins in lysosome-derived organelles; Palmer et al., 2013).

While only a few patients have been described with homozygous loss of *GRN* (Huin et al., 2020), the genetic and pathological link to NCL confirmed a previously suspected role for GRN in the lysosome. Earlier studies had already reported the transcriptional co-regulation of *GRN* and other lysosomal genes (Belcastro et al., 2011), and GRN was known to be rapidly endocytosed and delivered to the lysosome by the sorting receptor sortilin (SORT1) (Hu et al., 2010). The connection to NCL urged a reevaluation of brain tissue from previously described homozygous *Grn* knockout mice, revealing lipofuscin deposits and enlarged lysosomes, consistent with NCL (Ahmed et al., 2010; Smith et al., 2012; Wils et al., 2012). Consecutive studies demonstrated that GRN haploinsufficiency in humans similarly leads to features of NCL. Heterozygous *GRN* mutation carriers were shown to display preclinical retinal lipofuscinosis (Ward et al., 2017). At the neuropathological level, FTD brains demonstrate, in addition to TDP-43 proteinopathy, subtle features of NCL such as increased lipofuscinosis, intracellular NCL-like storage material as well as elevated expression of lysosomal proteins (for example CTSD and LAMP1/2) (Gotzl et al., 2014; Ward et al., 2017). Unexpectedly, the brain tissue of a homozygous *GRN* mutation carrier was devoid of TDP-43 cytoplasmic inclusions, although the loss of normal TDP-43 staining was seen in the nucleus of some neurons (Huin et al., 2020). Consistent with these findings, some NCL patients demonstrated increased phosphorylated TDP-43, although it remained largely soluble (Gotzl et al., 2014). This rare and surprising association of *GRN* mutations in NCL provided new insights into the pathological mechanisms underlying *GRN*-linked neurodegeneration and further broadened the therapeutic window.

### GRN as a Therapeutic Target in Neurological Diseases

The neuroprotective and immune modulatory roles of GRN have encouraged the therapeutic overexpression of GRN in a number of preclinical models for neurodegeneration.

In Alzheimer’s disease (AD), GRN cerebrospinal fluid (CSF) levels rise with disease progression and correlate with cognitive impairment and with the expression of the microglia derived soluble TREM2 (triggering receptor expressed on myeloid cells 2) (Suárez-Calvet et al., 2018). The rise in GRN is believed to reflect microglial activation in response to neurodegeneration. In AD mouse models, loss of GRN exacerbated plaque load and cognitive deficits, while lentiviral overexpression improved spatial memory and prevented hippocampal neuronal loss (Minami et al., 2014).

In a mouse model of motor neuron disease, increased microglial GRN expression marked the degenerative process (Philips et al., 2010). Human GRN overexpression was shown to rescue motor neuron abnormalities in zebrafish caused by mutant TDP-43 (Laird et al., 2010), but failed to affect a mutant SOD1 phenotype in zebrafish and mice (Laird et al., 2010). In mice, similar effects were observed: GRN overexpression could reduce the accumulation of insoluble TDP-43, slow down the axonal loss and improve survival of mutant TDP-43 mice, but was ineffective in mutant SOD1 mice (Herdewyn et al., 2013; Beel et al., 2018). Motor neuron defects in zebrafish caused by FUS mutants or by loss of SMN1, could be rescued by GRN as well (Chitramuthu et al., 2010, 2017).

The striatum is typically affected in GRN mutation carriers (Mackenzie, 2007), consistent with the high prevalence of Parkinsonism in these patients (Le Ber et al., 2008). In accordance, GRN has been shown to support the health of striatal neurons. In a mouse model of Parkinson’s disease, lentiviral *Grn* overexpression bolstered the resilience of nigrostriatal neurons to acute MTPT toxicity (Van Kampen et al., 2014), while loss of *Grn* rendered neurons vulnerable to toxicity (Martens et al., 2012). Additionally, neuroprotective effects of GRN have been demonstrated in ischemic brain injury. Intracerebroventricular administration of recombinant Grn two hours after ischemic brain injury was shown to reduce infarction volume and brain edema in a mouse stroke model (Egashira et al., 2013).

**Figure 2:**
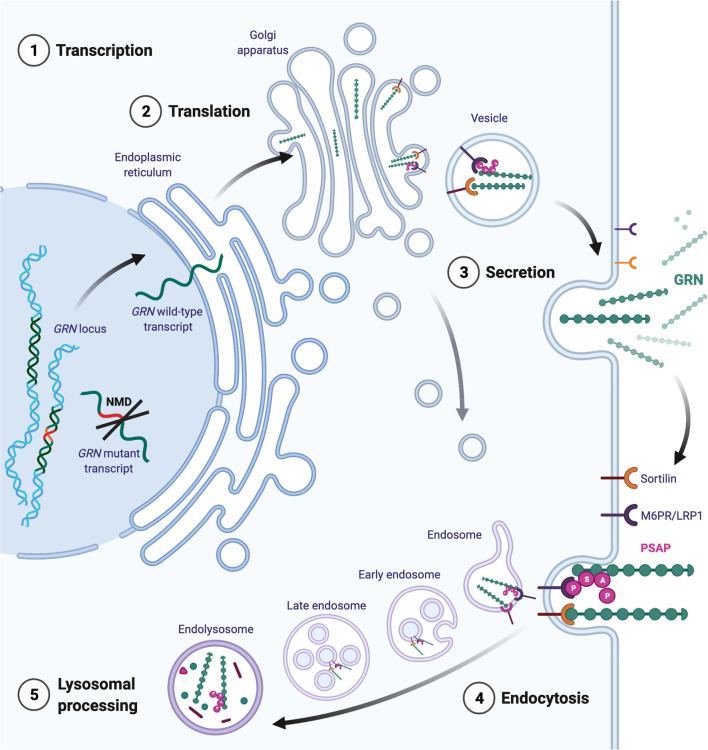
Intracellular trafficking of GRN. Transcripts from the mutated *GRN* allele are generally removed by nonsense-mediated mRNA decay (NMD). Transcripts from the healthy allele are translated directly into the endoplasmatic reticulum, where chaperones ensure proper folding. Mature GRN can be secreted constitutively or in an activity- dependent manner (for example in neurons). Extracellularly, GRN initiates tissue- and cell-type-dependent autocrine and paracrine signaling cascades. GRN binding partners are omitted for simplicity. Extracellular GRN levels are regulated through proteolytic degradation and by endocytosis. The binding of GRN to sortilin facilitates the uptake of GRN to endosomes. Alternatively, GRN can traffic to the endosome by indirect binding of the mannose-6-phosphate receptor (M6P) *via* prosaposin (PSAP). Additionally, GRN is thought to travel directly to the endosomal system *via* the trans-Golgi network.

These studies demonstrate the therapeutic potential of GRN as a potent modulator of neuroinflammation and neurotrophic factor in acute and chronic neurological disorders. The protective effects of GRN extend far beyond the neuropathology associated with *GRN* mutations. In the next paragraphs, we will discuss the complex cellular regulation of GRN expression and the associated therapeutic interventions. An overview of the life-cycle of GRN is provided in Figure 2.

## Transcriptional Mechanisms to Increase *GRN* Gene Expression

### Preventing Nonsense-Mediated mRNA Decay (NMD)

**Figure 3:**
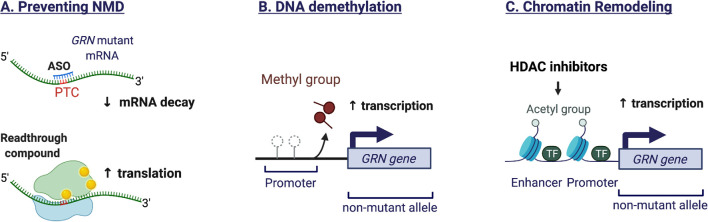
Mechanisms to increase endogenous *GRN* expression. **(A)** The majority of *GRN* mutations are nonsense and frameshift mutations that introduce premature termination codons (PTC). The presence of a PTC triggers the nonsense-mediated mRNA decay (NMD) machinery, resulting in degradation of the aberrant transcript and preventing protein translation. Antisense oligonucleotides (ASO) can stabilize the mutant mRNA by preventing the recruitment of NMD proteins. Alternatively, readthrough compounds can suppress nonsense mutations, leading to increased mRNA stability and protein translation. The efficacy of these approaches is mutation-dependent. **(B)** Epigenetic mechanisms regulate the expression of *GRN* from the healthy allele. *GRN* promotor demethylation promotes transcription. **(C)** Histone acetylation favors an open chromatin structure that is more permissive to gene transcription. HDAC inhibitors can prevent histone deacetylation and therefore increase *GRN* expression.

In patients with *GRN* mutations, transcripts from the mutated allele are generally removed by nonsense-mediated mRNA decay (NMD), as the majority of *GRN* mutations are nonsense and frameshift mutations that introduce premature termination codons (PTC). One therapeutic option could therefore be to block NMD in order to salvage truncated transcripts or to promote read-through of nonsense codons. The efficacy of these approaches hinges on whether the proteins resulting from nonsense suppression retain substantial function. A study in rodents, focusing on the most prevalent nonsense mutation (GRNR493X), provided proof of principle for the inhibition of NMD by use of antisense oligonucleotides (ASO) as well as by genetic and pharmacological intervention (Nguyen et al., 2018). The *GRN* R493X truncation mutant, lacking 17 percent of the protein, retained the ability to suppress the expression of inflammatory markers and influenced gene expression (Nguyen et al., 2018). In human *GRN* mutant iPSC-derived cortical neurons and astrocytes, a novel class of aminoglycoside PTC readthrough enhancer compounds increased GRN expression and improved lysosomal function (Frew et al., 2020). As the compounds did not readily cross the blood-brain barrier, the authors used intracerebroventricular delivery in a rodent model, to provide *in vivo* efficacy. Preventing NMD *via* antisense oligonucleotide-based approaches or pharmacological compounds thus seems feasible, albeit tailored to a selective number of pathogenic *GRN* mutations. Figure 3 illustrates the mechanisms to increase *GRN* gene expression.

### Stimulation of Endogenous *GRN* Expression

Very little is known about the physiological regulation of *GRN* expression and the differences between cell types. In the brain, *GRN* is primarily expressed by neurons and microglia. While microglia can upregulate *GRN* expression when activated, neurons maintain a stable GRN expression, only to increase with age (Petkau et al., 2010). Candidate epigenetic mechanisms are gene inactivation by methylation of the *GRN* promoter region or by limiting the accessibility of chromatin by histone deacetylation.

Evidence for the epigenetic regulation of *GRN* expression was found in sporadic patients with FTD. Compared to controls as well as patients with Alzheimer’s and Parkinson’s disease, sporadic FTLD brains exhibited altered expression of the DNA methyltransferase 3a as well as hypermethylation of the *GRN* gene promoter. Chronic treatment of *GRN* mutant lymphoblast lines with a methyltransferase inhibitor could rescue *GRN* transcript and protein levels in the study (Banzhaf-Strathmann et al., 2013).

In another attempt to influence the transcription of the healthy *GRN* allele in patients, Cenik and colleagues assessed *GRN* promotor activity in a luciferase-based screen. They identified the histone deacetylase inhibitor suberoylanilide hydroxamic acid (SAHA) as an enhancer of *GRN* expression and validated their results in cells from patients with frontotemporal dementia (Cenik et al., 2011). SAHA is currently in use as a treatment for cutaneous T-cell lymphoma but has significant side effects and limited brain penetrance (Khan and La Thangue, 2008). In December 2014 a phase 2a clinical trial was launched to assess the safety, tolerability, and pharmacodynamics of the highly brain penetrant HDAC inhibitor FRM-0334 (ClinicalTrials.gov identifier: NCT02149160). The compound induced GRN in rodent brain and in FTD-*GRN* patient-derived lymphoblasts but failed to increase GRN levels in patients at the doses tested (Boxer et al., 2019). Of note, the HDAC inhibitor valproic acid, frequently used as a mood stabilizer and for the treatment of epilepsy, was shown to increase *GRN* expression as well, notwithstanding the compound is less potent than SAHA (She et al., 2017). While HDAC inhibitors remain an interesting therapeutic option to increase *GRN* expression, their lack of specificity as general transcriptional enhancers is potentially problematic. An in-depth analysis of HDAC inhibitor binding kinetics and structural features required for the upregulation of *GRN* expression is provided by Moreno-Yruela et al. (2019).

Focusing on known autophagy-lysosome modulators, the compound trehalose was identified as a potential therapeutic target. Trehalose, a natural disaccharide, increased *GRN* mRNA as well as intracellular and secreted GRN in *GRN* mutant iPSC-derived cortical neurons (Holler et al., 2016). To improve blood–brain-barrier penetrance, novel trehalose derivatives are currently in development (Holler et al., 2016). The importance of using human model systems in the identification of potential therapeutic agents was demonstrated in a trial investigating the calcium channel blocker Nimodipine. In a rodent model system, the compound was shown to increase GRN levels but in *GRN* mutation carriers, Nimodipine failed to significantly raise GRN CSF levels (Sha et al., 2017).

### Exogenous *GRN* Expression

Gene therapy, the *in vivo* delivery of exogenous DNA, could be an alternative to influencing endogenous gene expression. Recombinant AAV delivers cargo DNA to the nucleus, but in contrast to the native AAV, does not integrate the transgene in the genome. The transgene persists as an episome in the nucleus of the transduced cell (Naso et al., 2017). Expression of *Grn* in neurons *via* adeno-associated viral delivery was able to correct neuropathological and behavioral abnormalities in heterozygous and homozygous *Grn* mutant mice. Interestingly, the vector carrying the *Grn* gene was only introduced after the onset of pathology in aged mice (10–12 months of age; Arrant et al., 2018). However, in a different study, T cell-mediated hippocampal degeneration was reported as a consequence of adeno-associated viral delivery of the *Grn* gene (Amado et al., 2019). The group used a different AAV9 strain, an alternative (intraventricular) route of transgene delivery and analyzed the animals at a later time point (Amado et al., 2019). In order to facilitate the translation of AAV-based gene therapy to *GRN* haploinsufficient FTD patients, Hinderer *et al*., supported by biotech company Passage Bio, have evaluated several *GRN*-expressing AAV vectors in rhesus macaques by direct intra-cisterna magna delivery (Hinderer et al., 2020). Despite a transduction efficiency of less than 1 percent of neurons after a single administration, a prolonged increase of GRN levels was seen, up to 10-fold (and even 40-fold for AAV1) compared to normal human GRN CSF levels. Neuropathological examination of the animals showed no signs of hippocampal degeneration but revealed mild axonal degeneration of sensory neurons in the posterior columns of the spinal cord, a side effect previously reported in other AAV studies using different transgenes (Hinderer et al., 2020). These results have supported the development of an AAV1-*GRN* treatment, called PBFT02, which will be studied in a Phase 1 study. Passage Bio plans to conduct the phase 1 trial in 2021 to assess the safety, tolerability, and efficacy of PBFT02 (ClinicalTrials.gov Identifier: NCT04747431). In July 2020, Prevail Therapeutics started the first *GRN*-AAV Phase 1/2 clinical trial in 15 people with FTD due to a *GRN* mutation. The study evaluates a different *GRN*-AAV9 vector, administered in combination with immunosuppressive drugs (PR006, ClinicalTrials.gov Identifier: NCT04408625).

As an alternative to gene therapy, direct intracerebroventricular delivery of recombinant GRN could allow a more controlled and dosed delivery of GRN. Whereas this delivery method has been safely tested before for other recombinant proteins (Van Damme et al., 2020), it still remains an invasive and expensive method of drug delivery.

**Figure 4:**
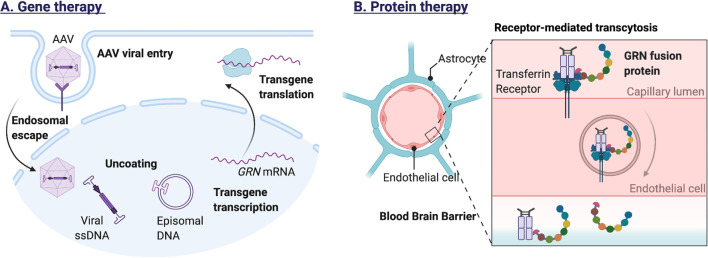
Methods for exogenous GRN expression. **(A)** Adeno-associated viral vectors, modified to carry the *GRN* gene, can infect a wide range of cells in the central nervous system. Recognition by cell surface receptors of the host cell triggers endocytosis and endosomal escape of the AAV particle. After nuclear import, the viral DNA is released, allowing transgene expression. Because the viral DNA persists as a nonintegrating stable episome, transgene expression can be prolonged, especially in non-dividing cells (e.g., neurons). **(B)** To allow intravenous protein therapy, a recombinant human GRN protein has been developed with improved blood–brain-barrier (BBB) penetrance. The GRN fusion protein binds the human transferrin receptor, facilitating receptor-mediated transcytosis across the blood-brain barrier.

To allow intravenous protein therapy, a recombinant human GRN protein has been developed with improved blood–brain-barrier (BBB) penetrance. The protein named PTV:PGRN (Protein Transport Vehicle) was engineered to bind to the human transferrin receptor, facilitating transport across the BBB. Preliminary reports state that intravenously administered PTV:PGRN can correct inflammatory and lysosomal lipid alterations in *Grn* null mice (Logan et al., 2020). Figure 4 illustrates the methods for exogenous GRN expression.

## Translational Control of GRN Expression

### Ribosomal Stalling

Research conducted by Capell et al. (2014) provided insights into the translational regulation of *GRN* expression. The group identified different *GRN* transcripts with short and long 5′ untranslated regions (UTR). Expression of the long 5′UTR, containing an upstream open reading frame, stalled the ribosomal translation of *GRN* and affected mRNA stability. Similar repression mechanisms by 5′UTRs have been demonstrated for the mRNAs of BACE1 and ADAM10. Selective repression of *GRN* translation could contribute to the differential expression of for example neurons and microglia, although to our knowledge, this has not been investigated.

### Target mRNA Degradation

Several microRNAs have been identified in the past years that can affect *GRN* mRNA stability and translation. The 3′UTR of progranulin contains a predicted miR-29b binding site and the knockdown of miR-29b was shown to increase the secretion of GRN in NIH3T3 cells (Jiao et al., 2010). *GRN* was identified as the strongest target for mIR-107 in an RNA immunoprecipitation technique coupled to microarray. Interestingly the expression of mIR-107 itself was glucose-sensitive and mIR-107 is downregulated in Alzheimer’s disease (Wang et al., 2010). A third miRNA affecting GRN expression was discovered when sequencing the *GRN* gene of more than three hundred FTLD patients with no known *GRN* mutation. A single-nucleotide polymorphism (SNP), rs5848, in the 3’UTR of the *GRN* gene was overrepresented in FTLD cases and was predicted in silico to increase the binding of miRNA-659, resulting in decreased GRN expression (Rademakers et al., 2008). However, in a different FTLD cohort, the SNP rs5848 risk allele was not reported (Simon-Sanchez et al., 2009). The recent finding that the risk allele is specifically associated with pathological subtype type A of FTLD-TDP and not with other subtypes (type B or C) could explain these differences (Pottier et al., 2019). Interestingly, the same SNP rs5848 has been described as a risk factor for Alzheimer’s disease and as a disease modifier for ALS as well (van Blitterswijk et al., 2014). Presence of the rs5848 risk allele was associated with decreased survival after onset in carriers of a C9orf72 repeat expansion.

Targeting miRNAs could be a potential therapeutic approach to recover GRN expression. To achieve miRNA inhibition, engineered oligonucleotides can be developed to mask the miRNA target sequence in the 3′ UTR of the target gene, preventing the miRNA/mRNA interaction. Alternatively, oligonucleotides can be developed that interfere directly with mature miRNAs and consequently compete with target genes, preventing target gene mRNA destruction (Atri et al., 2019).

### RNA-Binding Proteins

**Figure 5:**
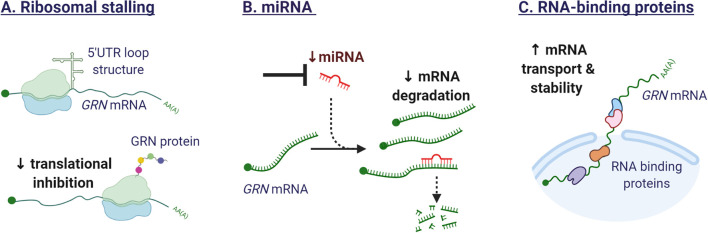
Mechanisms affecting GRN translation. **(A)** The secondary structure of the 5′ untranslated regions (UTR) in the *GRN* transcript can affect ribosomal function. The formation of a loop structure can hinder efficient translation. **(B)** Several miRNA have been identified that target *GRN mRNA* and repress GRN protein synthesis. Synthetic oligonucleotides could sequester endogenous miRNAs or mask miRNA target sequences in the *GRN mRNA*, leading to enhanced gene expression. **(C)** RNA-binding proteins are potential therapeutic targets as they affect the splicing, mRNA transport, stability, and translation of *GRN mRNA*.

Shortly after the discovery of mutations in the *GRN* gene, TDP-43 was identified as the key constituent of the tau-negative neuronal inclusions (Neumann et al., 2006). TDP-43 is an RNA-binding protein, involved in pre-mRNA splicing, mRNA transport, stability, and translation (Tziortzouda et al., 2021). As an RNA-binding protein, TDP-43 was suspected to regulate *GRN* levels. The relationship between TDP-43 and GRN however, is not straightforward. While studies have demonstrated that TDP-43 binds to the 3′ untranslated region of *GRN* mRNA, promoting mRNA instability (Colombrita et al., 2012; Fukushima et al., 2019), GRN protein levels were not decreased. The observed difference in transcript and protein levels could be explained by the concurrent effect of TDP-43 on *SORT1* mRNA (Gumina et al., 2019). Depletion of TDP-43 in human cells was found to increase the production of a splice variant of sortilin, that is secreted and not bound to the plasma membrane (Prudencio et al., 2012), impeding the normal uptake and delivery of GRN to the lysosome (Hu et al., 2010). The identification of additional RNA-binding proteins that regulate the stability of the *GRN* transcript, would provide novel therapeutic targets to modulate *GRN* gene expression. Figure 5 illustrates the mechanisms influencing GRN translation.

## Post-Translational Regulation of GRN Expression

### GRN Protein Structure

An N-terminal signaling sequence directs the nascent GRN protein chain to the endoplasmatic reticulum, where the protein undergoes extensive post-translational modifications. Proper protein maturation is essential for the biological activity of GRN and includes steps of signal sequence cleavage, the addition of *N*-linked glycans, and protein folding by chaperones in the lumen. Full–length GRN consists of 593 amino acid residues with a predicted molecular weight of 68.5 kDa, but an observed mass of approximately 75–80 kDa due to heavy glycosylation (Bateman and Bennett, 1998). Fucosylated oligosaccharides are the major *N*-linked glycans found in GRN (Songsrirote et al., 2010). The regulation and functional consequences of the different glycosylation sites are currently poorly understood.

Full-length GRN consists of seven and a half cysteine-rich granulin modules. Each granulin module folds into a prototypic structure of four β-hairpins, joined together by six disulfide bridges (He and Bateman, 2003). The proper folding and transport of GRN through the ER-Golgi pathway is assured by several ER Ca2+-binding chaperones including calreticulin, Binding immunoglobulin protein (BiP), GRP94, and members of the protein disulfide isomerase family (Almeida et al., 2011). Modulating the ER chaperone network has been proposed as a therapeutic strategy to increase the level of extracellular GRN.

### GRN Secretion

The presence of an amino-terminal secretion signal directs mature GRN through the trans-golgi network into secretory vesicles, in which it undergoes regulated exocytosis (Petoukhov et al., 2013). GRN can be secreted as a soluble protein or associated with exosomes (Benussi et al., 2016). Alternatively, by binding sortilin or through an interaction with prosaposin, GRN can be diverted from the secretory pathway to the endolysosomal system (Hu et al., 2010; Zhou X. et al., 2015). The interaction between GRN and prosaposin and sortilin will be discussed further in the next section.

In neurons, GRN was found to be co-transported with BDNF and recruited to synapses for secretion in an activity-dependent matter (Petoukhov et al., 2013). Alkalizing drugs and vacuolar ATPase inhibitors (like chloroquine or bafilomycin) can increase GRN levels within the secretory pathway, with increased levels in the ER and the Golgi network as well as extracellular (Capell et al., 2011). Chloroquine is commonly used as an anti-malarial drug and to treat autoimmune diseases. In an exploratory clinical study, three FTD patients known to be GRN deficient, received chloroquine treatment for a year, with a reported rescue of GRN CSF levels and a reported improvement in cognitive abilities (Körtvelyessy et al., 2017). However, to our knowledge, there are currently no clinical trials further investigating chloroquine as a treatment option for FTD.

## Regulators of Extracellular GRN Levels

### Sortilin

While mutations in *GRN* can be predicted based on a reduced blood GRN levels, this does not explain the large variability in plasma GRN levels seen in healthy controls (Finch et al., 2009; Meeter et al., 2016). Genetic studies have therefore sought to reveal additional regulators of GRN expression. A repurposed genome-wide association study (GWAS) of late-onset Alzheimer’s Dementia, revealed two SNPs on chromosome 1 linked to lower plasma GRN levels. The most significant SNP, rs646776, was associated with a modest reduction (16 percent) in plasma GRN levels and increased expression of the sortilin gene (*SORT1*) (Carrasquillo et al., 2010). Sortilin, a lysosomal sorting and trafficking receptor, was simultaneously identified in a screen for GRN binding partners using monkey kidney cells (unable to bind GRN) overexpressing a cDNA library (Hu et al., 2010). Knocking out *SORT1* severely reduced the binding of GRN to the cell surface with a concomitant rise in extracellular levels of GRN (Hu et al., 2010).

Initially, sortilin was thought to be the long sought-after GRN receptor, mediating the neurotrophic effect. Sortilin was known to interact with the low-affinity neurotrophin receptor p75NTR and mediate proneurotrophin-induced apoptosis (Nykjaer et al., 2004). In addition, the extracellular part of sortilin binds granulin E (Zheng et al., 2011), the most C-terminal part of GRN and a granulin known to exert neurotrophic properties on cortical and motor neurons (Van Damme et al., 2008). However, despite the deletion of the sortilin binding site, granulin E was capable of promoting the survival of mouse motor and cortical neurons (De Muynck et al., 2013). Likewise, GRN could still stimulate neurite outgrowth in hippocampal cultures of *Sort1* null mice, suggesting GRN acted independently of sortilin (Gass et al., 2012).

Nevertheless, the identification of sortilin as a receptor and regulator of GRN levels, did instigate the therapeutic targeting of the GRN-sortilin axis. As sortilin targets GRN for destruction in the lysosome (Hu et al., 2010), preventing GRN uptake and destruction could protect neurons and improve disease. Small molecule MPEP, known to decrease sortilin levels, has been shown to increase extracellular GRN levels but not intracellular GRN in iPSC-derived neurons harboring the *GRN* S116X mutation (Lee et al., 2014). A different tactic is the development of a sortilin antibody, a therapeutic approach currently pursued by Alector in partnership with Abbvie. AL001, a monoclonal human recombinant anti-human sortilin antibody, has progressed to Phase 2 and Phase 3 studies (ClinicalTrials.gov Identifier: NCT03987295 and NCT04374136). The completed Phase 1 study (NCT03636204) demonstrated that AL001 treatment could normalize GRN CSF levels in symptomatic and asymptomatic *GRN* mutation carriers (Haynes et al., 2020). Eight weeks after the last dose, a modest reduction of plasma neurofilament light chain (NfL) was observed, hinting at a possible beneficial effect on neuronal integrity. NfL, a neuron-derived cytoskeletal protein, is a promising biomarker of neurodegeneration and has been shown to increase over time in symptomatic *GRN* mutation carriers (van der Ende et al., 2019).

At the cellular level, the impact of redirecting GRN *via* inhibition of sortilin is incompletely understood. A number of studies have recently accentuated the role of full-length GRN and granulin peptides in the lysosome (Holler et al., 2017; Lee et al., 2017; Zhou et al., 2017a). Inhibiting the endocytosis of GRN could therefore further deplete GRN and the granulins in the lysosome, possibly affecting lysosomal homeostasis. Then again, blocking sortilin could increase the availability of GRN to alternative receptors (e.g., the EphA2 receptor) (Neill et al., 2016). This duality underscores that continued mechanistic research is needed, in parallel with the development of therapies controlling GRN levels.

### Prosaposin

Linking whole-genome sequencing data with plasma GRN levels in a large Mexican-American cohort, led to the confirmation of prosaposin (PSAP) as a regulator of GRN (Nicholson et al., 2016). GRN and prosaposin were known to be structurally and functionally very similar and the interaction between GRN and prosaposin had been identified through a proteomic screen, one year prior (Zhou X. et al., 2015). Prosaposin can, like progranulin, be cleaved into cysteine-rich subunits called saposins and homozygous loss-of-function mutations lead to dysfunction in lysosomal sphingolipid metabolism (sphingolipidosis) (Zhou X. et al., 2015). Prosaposin binds the mannose-6-phosphate receptor (M6PR) or low-density lipoprotein receptor-related protein 1 (LRP1) to gain access to lysosomes. By forming heterodimers, GRN and prosaposin were shown to use each other’s transport receptors to travel to the lysosome (Zhou X. et al., 2015; Nicholson et al., 2016). The finding provided a new perspective on the role of sortilin as a GRN signaling receptor. Could GRN have stimulated neurite outgrowth and survival independent of sortilin (Gass et al., 2012; De Muynck et al., 2013), because there is a functional interchangeability of the trafficking receptors in the presence of prosaposins? The functional redundancy could indicate a fail-safe mechanism, underscoring the importance of the pathway.

### TMEM106B

An international collaboration identified variants on chromosome 7p21, associated with increased TMEM106B expression, as susceptibility loci for FTLD-TDP (Van Deerlin et al., 2010). Interestingly, the association of the risk locus was stronger in *GRN* mutation carriers (Van Deerlin et al., 2010). Later it was shown that the risk locus correlated with decreased plasma GRN levels (Finch et al., 2011) and elevated TMEM106B protein levels in patient FTLD brain samples (Chen-Plotkin et al., 2012). The clinical importance of variants in TMEM106B for *GRN* mutation carriers was recently demonstrated by Pottier et al. (2018). Carriers of the protective haplotype, associated with lower levels of TMEM106B, are estimated to have 50% less chance of developing disease symptoms (Pottier et al., 2018). In ALS patients, the protective allele was associated with preserved cognition (Vass et al., 2011). The role of the lysosomal transmembrane protein TMEM106B as an aggravating risk factor has been explored and a reciprocal regulation between GRN and TMEM106B levels has become evident. GRN deficiency leads to increased TMEM106B levels in aged mice (Zhou et al., 2017b). Overexpression of TMEM106B in a cell model caused abnormalities in late endosome-lysosome morphology and led to the sequestration of GRN (Chen-Plotkin et al., 2012).

Complete loss of *Tmem106b* however, was not beneficial in *Grn* knockout mice models (Feng et al., 2020; Werner et al., 2020; Zhou et al., 2020). Double knockout mice demonstrated worsened neuropathology with increased TDP-43 accumulation, gliosis, and lysosomal dysfunction. Additionally, the mice developed motor deficits, a phenotype not present in single knockout models (Feng et al., 2020; Werner et al., 2020; Zhou et al., 2020). In knockout models that retained a low residual expression of *Tmem106b*, a phenotypic delay was seen with motor deficits emerging 2 months later (Zhou et al., 2020). Residual *Tmem106b* expression could explain the partial rescue phenotype observed by Klein et al. (2017). In that study, removing *Tmem106b* reversed neuronal death and a hyperactive behavioral phenotype associated with complete loss of *Grn*. Both *Tmem106b* deletion (Klein et al., 2017) as well as overexpression impaired lysosome acidification (Chen-Plotkin et al., 2012). While the possibility to manipulate TMEM106B levels remains an interesting new therapeutic avenue, these functional studies indicate the importance of accurately balancing TMEM106B levels. One potential additional drawback to manipulating TMEM106B is the identification of *de novo* mutations as a rare cause for hypomyelinating leukodystrophy (Simons et al., 2017). Further study is needed to fully gauge the consequences of the TMEM106B variants in *GRN*-FTLD and to determine how GRN and TMEM106B interact.

## GRN Processing

### Extracellular Processing

**Figure 6:**
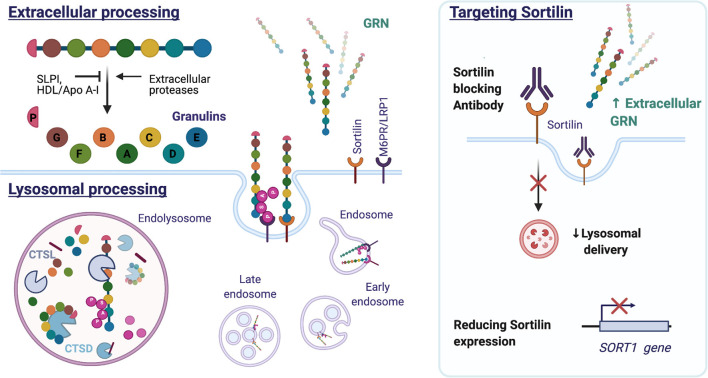
Structure and processing of mammalian GRN. GRN consists of seven and a half granulin domains. The linker sequences can be cleaved by extracellular and intracellular proteases, releasing individual granulins or a combination of GRN modules. Both SLPI and HDL/Apo A-I can bind GRN and inhibit GRN processing. Extracellular GRN is rapidly endocytosed and targeted to the lysosome directly by binding sortilin or indirectly by forming a heterodimer with prosaposin, which utilizes the M6PR or LRP1 receptors. In the lysosome, GRN is cleaved into granulins by Cathepsin L (CTSL) and possibly other hydrolases. GRN contributes to lysosomal homeostasis by increasing the stability of lysosomal enzymes [e.g., Cathepsin D (CTSD) and consequently affects the processing of prosaposin (PSAP)]. Reducing sortilin (*SORT1*) gene expression or preventing GRN-sortilin interaction with blocking antibodies can increase extracellular GRN levels and prevent lysosomal degradation of GRN (HDL: high-density lipoprotein, Apo A-I: apolipoprotein A-I, M6PR: mannose 6-phosphate receptor, LRP1: lipoprotein receptor-related protein 1).

Extracellular GRN levels are regulated through endocytosis and proteolytic degradation. Individual six kDA granulins can be excised from the full-length GRN protein by extracellular proteases, for example, metalloproteinases MMP-9, MMP-12, MMP-14, and ADAMTS-7 (De Muynck and Van Damme, 2011). Both full-length GRN and the released granulin peptides are biologically active but the cellular effects can differ from granulin to granulin and can often antagonize the actions of full-length GRN (Bateman and Bennett, 2009). The granulins were in fact identified before the discovery of the intact precursor protein, in extracts of granulocytes, hence the origin of the name granulins (Bateman et al., 1990). They were later shown to possess pro-inflammatory properties and to negatively impact wound healing in contrast to their common precursor (Zhu et al., 2002). Granulins regulate cell growth but with opposing effects as demonstrated on breast cancer cell lines for GRN A and GRN F (Tolkatchev et al., 2008; Bateman and Bennett, 2009). Granulin E, the most C-terminal part of full-length GRN exerts neurotrophic properties on cortical and motor neurons (Van Damme et al., 2008). The precursor protein is protected from cleavage by association with the high-density lipoprotein (HDL)/Apolipoprotein A-I complex (Okura et al., 2010), or by binding to the secretory leukocyte protease inhibitor (SLPI; Zhu et al., 2002). Figure 6 illustrates the intracellular and extracellular processing of the GRN protein.

### Lysosomal Processing

Extracellular GRN is rapidly endocytosed and delivered to the lysosome by SORT1 (Hu et al., 2010) or in association with prosaposin (PSAP) (Zhou X. et al., 2015). While the lysosome was previously considered a degradation endpoint for GRN, research attention is currently focused on probing the lysosomal function of the protein. Cathepsin L, a lysosomal cysteine protease, was found responsible for the lysosomal processing of GRN (Holler et al., 2017; Lee et al., 2017; Zhou et al., 2017a). Excised granulins were shown to be more stable and to persist in the lysosome long after endocytosis and the disappearance of full-length GRN (Holler et al., 2017). The function of granulins in the lysosome remains to be further elucidated (reviewed in Zhou et al., 2021).

Apart from being a substrate of lysosomal enzymes, full-length GRN functions as a chaperone to lysosomal hydrolases like cathepsin D and β-glucocerebrosidase (Jian et al., 2016; Beel et al., 2017). Interestingly, mutations in GBA1, the gene encoding β-glucocerebrosidase, are common genetic risk factors for the development of Parkinson disease, and homozygous mutations are associated with Gaucher disease, a juvenile onset lysosomal storage disease (Paushter et al., 2018).

Selective inhibition of GRN proteases could be of great therapeutic value, but requires a better understanding of GRN protein catabolism.

## GRN Signaling Receptors

Growth factor signaling cascades are known to be important for neuronal survival and function, but GRN turns out to be far from a conventional growth factor. While GRN activates classical growth factor signaling cascades, including the mitogen-activated protein (MAP) kinase and phosphoinositide 3 (PI3) kinase pathways, it does not bind a classical growth factor receptor nor does the mode of growth factor signaling resemble that of other growth factors like PDGF or EGF (Bateman and Bennett, 2009). In IGFI-R-deficient fibroblasts, GRN was able to elicit a prolonged growth factor signal that carried the fibroblast through both S phase (DNA synthesis phase) and M phase (cell division phase) (Zanocco-Marani et al., 1999). GRN is therefore simultaneously both a competence and a progression factor, which is a unique feature as classical growth factors normally require the assistance from other growth factors to complete both S- and M-phase. In neurons, the MAP kinase pathway and PI3K pathways are involved in GRN-dependent neuroprotection (Xu et al., 2011; Almeida et al., 2012). Apart from evoking classical growth factor signaling cascades, GRN additionally promotes tyrosine phosphorylation of focal adhesion kinase (FAK) (He and Bateman, 2003). FAK is a critical mediator of integrin signaling and could therefore potentially explain GRN’s influence on cell migration, cell cycle progression, and prevention of anoikis.

### TNF Receptor

In 2011, using a yeast-two-hybrid screen, GRN was reported as a new ligand and antagonist of tumor necrosis factor (TNF) receptors (Tang et al., 2011). The finding was not replicated, possibly due to technical differences and the conformation of the recombinant GRN protein used in the study (Chen et al., 2013). Later studies confirmed GRN as a ligand of TNFR, implicating GRN in many inflammatory diseases (e.g., contact dermatitis, osteoarthritis, colitis, etc.). Wang et al. (2015) provide an overview of the confirmation experiments and initial controversies regarding the GRN-TNFR interaction. Atsttrin, a more stable GRN-like engineered protein, was shown to suppresses TNF-α-mediated inflammation in arthritis models (Wei et al., 2017).

Additionally, GRN has been shown to interact with the toll-like receptor 9 (Park et al., 2011), a key regulator of the immune response to foreign DNA. GRN also bound the extracellular domain of Notch receptors and affected Notch target gene expression (Altmann et al., 2016). The Notch signaling system has distinct roles in innate immunity and cell development. DLK1, a modulator of Notch signaling, was found to interact with GRN in a yeast-two-hybrid screen (Baladron et al., 2002).

### EphA2

In 2016, EphA2 was identified as a functional high-affinity GRN receptor, using a phospho-RTK array and a human urinary bladder carcinoma cell line. Silencing the receptor or blocking the ligand interaction, affected capillary lumen formation by human umbilical vein endothelial cells (HUVEC) as well as *GRN* transcriptional autoregulation (Neill et al., 2016). Analysis of downstream signaling events [activation of MAPK and protein kinase B (Akt) pathways], suggested GRN antagonizes Ephrin/Eph signaling (Neill et al., 2016). Considering the known involvement of Ephrin signaling in axon guidance and synaptogenesis, the finding poses questions for future study. The same research group identified drebrin, an F-actin–binding protein, and perlecan, an ECM proteoglycan, as GRN binding partners (Gonzalez et al., 2003; Xu et al., 2015).

The interaction of GRN with a multitude of extracellular, transmembrane, intracellular, and nuclear proteins is remarkable (De Muynck and Van Damme, 2011; Cui et al., 2019; Zhou et al., 2021) However, the multitude of GRN binding partners is the result of researching GRN biology in diverse histological contexts (peripheral inflammation, vasculogenesis, carcinogenesis and so forth). Regarding GRN biology in the CNS, it seems likely that concerted actions of different binding partners contribute to the neurotrophic effect.

## Concluding Remarks

In recent years, significant progress has been made towards the development of therapies modulating GRN levels. While raising GRN has always seemed straightforward, the multitude of mechanisms at play in regulating GRN levels pose extra challenges for the development of therapeutic agents. With a common goal to raise GRN levels, every therapeutic intervention differs in the extent of action depending on the biological strategy used (e.g., affected by viral vector tropism or depending on *SORT1* expression) and by mode of administration (e.g., systemic or CNS restricted delivery). The resulting pattern of (ectopic) GRN expression will therefore influence the biological effect and possible side-effects. Close monitoring of GRN protein levels will be needed to ensure expression within a physiological range, as elevated GRN levels have been associated with cancer, metabolic disease, and chronic inflammatory states.

The current development and utilization of GRN therapy in FTD patients with *GRN* mutations provides a unique starting point for further research. The recent extensive documentation of fluid, neuroimaging and cognitive biomarkers for FTD will undoubtedly prove to be crucial in monitoring treatment response and guiding therapeutic development (Benussi et al., 2021; Panman et al., 2021). Additionally, specific biomarkers for the biological effects of GRN are needed to identify candidate diseases for GRN therapy. The pleiotropy of the GRN protein ensures that the applicability of GRN as a therapeutic target reaches far beyond the ALS-FTD spectrum.

## Author Contributions

JT, CV, and PVD wrote the manuscript. All authors made substantial contributions to the discussion of the content, reviewed and edited the article. All authors contributed to the article and approved the submitted version.

## Conflict of Interest

The authors declare that the research was conducted in the absence of any commercial or financial relationships that could be construed as a potential conflict of interest.

## Publisher’s Note

All claims expressed in this article are solely those of the authors and do not necessarily represent those of their affiliated organizations, or those of the publisher, the editors and the reviewers. Any product that may be evaluated in this article, or claim that may be made by its manufacturer, is not guaranteed or endorsed by the publisher.
